# Treatment Strategies of Adult Primary Focal Segmental Glomerulosclerosis: A Systematic Review Focusing on the Last Two Decades

**DOI:** 10.1155/2016/4192578

**Published:** 2016-04-07

**Authors:** Arno Beer, Gert Mayer, Andreas Kronbichler

**Affiliations:** Department of Internal Medicine IV (Nephrology and Hypertension), Medical University of Innsbruck, Anichstraße 35, 6020 Innsbruck, Austria

## Abstract

Adult primary focal segmental glomerulosclerosis (FSGS) remains a therapeutic challenge for the treating physician. With the advent of novel immunosuppressive measures, our arsenal of therapeutic options increased considerably. The aim of this review was to summarize reports published over the last two decades which reported on treatment outcome. Most reports included patients with a steroid-resistant (SR) disease course, yet the cohort with the highest unmet need, since persistent nephrotic range proteinuria is associated with a poor renal prognosis and portends a high risk of developing end-stage renal disease. While in first-line treatment, steroid treatment remains the recommended standard with an overall remission rate of 50% and higher, optimal treatment strategies for steroid-dependent/multirelapsing (SD/MR) and SR patients have to be defined. In both entities, calcineurin inhibitors showed good efficacy, while mycophenolate mofetil was less effective in SR cases compared to those with SD/MR. The same was true for rituximab, a monoclonal antibody targeting B-cells. In resistant cases, addition of extracorporeal treatment options or treatment with alkylating agents may be considered. To shape the future for treatment of FSGS, international collaborations to conduct larger clinical trials are needed to identify potential novel efficacious immunosuppressive or immunomodulatory therapies.

## 1. Introduction

The incidence of focal segmental glomerulosclerosis (FSGS) has increased over the past decades and it is assumed to be one of the leading causes of idiopathic nephrotic syndrome in adult patients. Racial disparities have been reported with African American being two to three times more often affected than Caucasian [1]. Despite an increased arsenal of therapeutic options, treatment of this glomerular lesion is remaining a challenge for nephrologists. In contrast to other primary forms of nephrotic syndrome spontaneous remission is rare (<5%) and initiation of immunosuppressive measures should be commenced once diagnosis is confirmed by renal biopsy. Presence of nephrotic syndrome (>3–3.5 g/d) portends a poor prognosis with 50% of patients progressing to end-stage renal disease (ESRD) 6–8 years after initial diagnosis, whereas patients with nonnephrotic proteinuria in particular have a favorable outcome. Those with massive nephrotic syndrome (proteinuria > 10 g/d) tend to have an even more aggressive disease course with half of the patients reaching ESRD after 3 years. Serum creatinine above 1.3 mg/dL (approximately 114 *μ*mol/L) was associated with a poorer prognosis than a preserved renal function [2]. Analysis of the United States Renal Data System revealed that FSGS is the leading cause of glomerulonephritis-associated ESRD in the United States [3], highlighting the importance of improved surveillance (diagnosis early in the disease course) and improved strategy options to overcome treatment unresponsiveness.

Thus, the aim of this systematic review was to summarize the literature published over the last two decades focusing on the treatment of adult primary FSGS.

## 2. Materials and Methods

To evaluate literature-based publications over the last two decades, the MEDLINE database search was restricted to a time period ranging from January 1995 to 31 October 2015. The search was conducted using the keywords “focal segmental glomerulosclerosis” AND “treatment”. Restricting the time frame to the last two decades would allow access to a majority of identified manuscripts.

We predefined the following exclusion criteria for further data analysis: (i) presenting data on recurrence of FSGS after kidney transplantation, (ii) combining treatment data of children and adults with a predominance of the former, (iii) reporting no concrete outcome data (i.e., not reporting on the number of patients achieving either partial/complete remission or treatment failures and/or not the mean decrease of proteinuria following initiation of treatment), (iv) no differentiation whether patients had an underlying primary FSGS or other entities leading to nephrotic syndrome, (v) no differentiation of treatment modalities (i.e., combining results of patients receiving prednisolone and other immunosuppressive measures in final analysis), (vi) no differentiation between steroid-dependent (SD)/multirelapsing (MR) and steroid-resistant (SR) patients in the analysis of the results, or (vii) reports including a low number of patients (arbitrary cut-off ≥ 5 patients).

## 3. Results

### 3.1. Search Strategy

**Figure 1:**
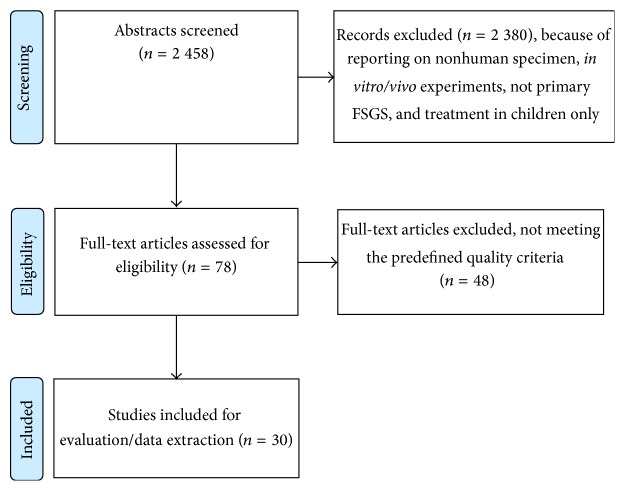
The search strategy “focal segmental glomerulosclerosis” AND “treatment” yielded a total of 2 458 abstracts which were evaluated regarding the predefined criteria. After initial evaluation, 78 articles were accessed in full text. Of these, 48 could be excluded due to not meeting the predefined criteria. Thus, data of 30 articles were extracted (modified from [4]: Preferred Reporting Items for Systematic Reviews and Meta-Analyses: The PRISMA Statement).

The systematic search resulted in an overall number of 2 458 records. A large number of articles (*n* = 2 380) could be excluded since these records reported on* in vitro* experiments, non-FSGS related studies, other entities leading to nephrotic syndrome, or findings in children. A total of 78 articles were left over after initial screening. After access of full text we could exclude another 48 articles, which did not meet our predefined inclusion criteria. Data were extracted from 30 articles reporting on treatment outcome of patients with primary FSGS (see Figure 1).

We divided the results obtained from the included studies into three categories, namely, first-line treatment, SD/MR, and SR.

### 3.2. First-Line Treatment Options in Focal Segmental Glomerulosclerosis

Most studies reported on first-line treatment consisting of daily oral prednisolone and in some cases in combination with other immunosuppressive measures. The total number of patients treated with prednisolone ranged from 8 to 79 patients in the respective studies. The overall response rate reported in these studies ranged from 50% [5] up to 68.8% in a prospective study conducted in India [10]. Follow-up of patients was highly diverse, ranging from 16.2 to 62 months. As expected, in the study with the shortest follow-up the relapse rate was the lowest (27.3%) [10], while Rydel and colleagues reported a relapse rate of 67% [5]. In the study reporting a single center experience, a majority received high dose prednisone for at least one month (87% ≥ 60 mg/d) and those remaining on high dose prednisolone treatment showed a significant trend towards better response. A multicenter retrospective analysis from Italy revealed remission of 31 out of 52 patients treated with steroids (either 1 mg/kg body weight for 8 weeks with subsequent tapering or three intravenous pulses of 1 g each followed by 0.5 mg/kg body weight oral prednisolone with subsequent tapering). All included patients had nephrotic range proteinuria measured at least twice ahead of treatment initiation. Of the 38 patients who did not achieve either complete or partial remission, 26 were retreated with either prolonged corticosteroid or other immunosuppressive measures (azathioprine, cyclosporine A, or cyclophosphamide). Among those receiving steroids two of the patients achieved complete and partial remission (out of six), while cytotoxic drugs and cyclosporine A (CSA) treatment led to one and zero complete as well as five and seven partial remissions (out of 11 and 9 patients) [6]. A study from Egypt included a total of 79 patients. Of these, a majority had nephrotic syndrome at the time of treatment. In total, 40 patients achieved remission followed induction treatment with prednisolone therapy (1 mg/kg body weight for 6 weeks, followed by 0.75 mg/kg body weight for another 6 weeks, and subsequent tapering). Mycophenolate mofetil (MMF) efficacy was evaluated in two studies. One retrospective cohort reported by Choi and colleagues treated patients either with or without concomitant steroid due to impaired renal function (*n* = 7) or nephrotic syndrome (*n* = 7). MMF dosage varied from 1.0 to 2.0 g per day and five out of ten patients achieved remission (3 complete and 2 partial). Follow-up was rather short with 7.9 months [7]. In a prospective study from India MMF (target dose 2 g/day for 6 months) was given along with a reduced steroid dose (0.5 mg/kg body weight as initial dosage, total treatment duration 2-3 months). Out of 17 patients, 70.8% achieved remission (10 complete and 2 partial). Remission rates were comparable to those receiving prednisolone monotherapy (initial dosage 1 mg/kg body weight, cumulative prednisolone dosage 7.3 ± 0.9 g versus 1.9 ± 0.3 g in the MMF group). Mild gastrointestinal discomfort was noticed in one patient and two patients in the MMF group required hospitalization due to severe infection. However, side effects were not reported independent of disease entity (patients with membranous nephropathy and FSGS). Subsequent relapse rate was similar in both groups as well [10]. Other studies reporting on first-line treatment in FSGS included a small number of patients only (<10). Among these, one reported on single center experience with tacrolimus monotherapy in six patients. All subjects achieved partial remission with a median reduction of proteinuria from 11 ± 4.5 to 2.8 ± 2.5 g/d, while serum albumin improved from 26.8 ± 4.6 to 37.7 ± 1.9 g/L. During a follow-up period of 12.8 months, no relapse was observed [8]. One study retrospectively analyzed patients treated with either prednisolone (1 mg/kg body weight) alone, prednisolone (0.5 mg/kg body weight) with azathioprine (AZA, 2 mg/kg body weight), or CSA (3 mg/kg body weight). Remission rates were higher in the latter two groups, whereas side effects were observed in the prednisolone group (3 patients became cushingoid) and leukopenia was observed in two patients being treated with AZA [9]. More details related to the single studies are highlighted in Table 1. Taken together, remission rates after steroid treatment are reported to be at least 50%. Alternative treatment strategies, such as MMF or tacrolimus, either in combination or as monotherapy, may yield similar remission rates. In patients with absolute or relative contraindication towards steroid treatment, these agents may have a role in the first-line treatment.

### 3.3. Steroid-Dependent/Multirelapsing Focal Segmental Glomerulosclerosis

We used “steroid-dependent” for patients achieving remission after steroid induction but having relapses upon steroid tapering or within two weeks after discontinuation [12], while we used “multirelapsing” for patients with a relapsing disease. Various studies/case reports investigated the effect of additional immunosuppressive agents on top of ongoing steroid treatment. In a prospective study from Korea, all patients (total *n* = 5) achieved remission (complete remission in 4 patients). At baseline all patients received daily oral prednisolone (total dosage 10 mg) with subsequent tapering and CSA in an initial dosage of 5 mg/kg body weight (with the aim of achieving a trough level between 100 and 200 ng/mL). If the trough level was not maintained above 100 ng/mL or patients had an incomplete response, CSA dose was increased up to 7 mg/kg body weight a day [13].

Experience with MMF is limited to one report including several entities leading to nephrotic syndrome. Among patients with FSGS, the median proteinuria decreased from 5.1 g/day at baseline to 1.9 g/day during follow-up. Remission was recorded in eight patients (3 complete and 5 partial), while 5 patients were nonresponsive towards MMF (target dose 1.5–2 g/day). Side effects were mainly restricted to gastrointestinal symptoms [14]. Sirolimus, targeting mammalian target of rapamycin (mTOR), was tested in a phase 2 open-label clinical trial. All patients (*n* = 7) had nephrotic range proteinuria at trial initiation. The target trough level during the first four months was 5–15 ng/mL and was further increased to 10–20 ng/mL during the following eight months. Therapy was stopped in five patients due to inefficacy and overall no patient experienced response to treatment [15]. Adrenocorticotropic hormone (ACTH) gel (80 units twice weekly) was tested in either SD or SR patients. Among those with SD FSGS, 2 patients showed a partial response (one with a not otherwise specified and one with a tip lesion histology), while four patients had no response (two not otherwise specified, one cellular, and one tip lesion). During follow-up, serum creatinine stabilized from a baseline median value of 1.5 mg/dL to 1.45 mg/dL (at follow-up), whereas serum album increased from 2.44 g/dL to 3.04 g/dL.

In their prospective trial, Ruggenenti et al. recruited patients with complete remission. The median relapse rate before and after rituximab (RTX) therapy was significantly reduced in the overall cohort. Among the eight patients with FSGS, three patients relapsed within a period of 12 months. In general, patients received a B-cell driven protocol (one infusion of 375 mg/m^2^, which was repeated when B-cells were present in peripheral blood after one week). Overall, the concomitant steroid use could be reduced and the authors did not report serious adverse events following B-cell depletion [16]. All reports included in our systematic review are summarized in Table 2. CSA and MMF may be useful measures in the treatment of patients with SD/MRFSGS and among the novel immunosuppressive measures, remission maintenance was achieved following RTX treatment in most patients. However, cohorts including larger numbers of patients treated in this indication are clearly needed to draw definite conclusions.

### 3.4. Steroid-Resistant Focal Segmental Glomerulosclerosis

In general, steroid-resistance is defined as persistence of proteinuria despite prednisone treatment (1 mg/kg body weight or 2 mg/kg body weight every other day) for at least 4 months [12]. Most reports over the last two decades have focused on treatment options in this cohort with the highest unmet needs. Calcineurin inhibitors have frequently been used in this indication. Overall response of patients receiving CSA ranged from 57.1% to 77.8% in the different studies [6, 17–20]. One randomized trial including 26 patients evaluated relapse rate after 24 months of follow-up. CSA was initiated with a dose of 3.5 mg/kg body weight with subsequent adaption to a trough level of 125–225 *μ*g/L, accompanied by a maximum prednisone dose of 15 mg with subsequent tapering over 26 weeks. Among those 18 patients with response (3 complete and 15 partial), 61% relapsed during the observational period. A decline in renal function defined as rise of 30% occurred in four patients. Increase in dosage or new prescription of an antihypertensive agent was necessary in eight patients [17]. A prospective study from Germany recruited patients failing a 6-week course of prednisolone (1.5 mg/kg body weight) and acetylsalicylic acid (500 mg/d). CSA was initiated with a trough level of 130–180 *μ*g/L. Serum creatinine was preserved (1.5 ± 0.2 mg/dL), while proteinuria was within nephrotic range (5.5 ± 2.6 g/d). Of the 34 patients, 8 (23%) and 13 (38%) of the patients achieved complete and partial remission, respectively [20]. Another controlled trial from Germany included nephrotic patients and showed a decline of proteinuria from 5.4 ± 5.2 g/d to 2.5 ± 1.8 g/d during a follow-up of three years. No concrete details related to duration of treatment or dosage were given [19]. There were two articles reporting a large number of patients with steroid-resistance receiving tacrolimus treatment. Interestingly, Segarra and colleagues recruited patients with either CSA-resistance or CSA-dependence and showed a high response rate following tacrolimus initiation (initial dose 0.15 mg/kg, with a targeted trough level of 5–10 ng/mL; overall remission rate 72%, 10 complete and 8 partial remissions) combined with prednisone (1 mg/kg body weight for 4 weeks, with subsequent tapering) [21], while a prospective study from India revealed an overall response of 52.3% (17 complete and 6 partial remissions) following treatment initiation of tacrolimus (0.1 mg/kg body weight; trough level 5–10 ng/mL) and oral prednisolone (0.15 mg/kg body weight). The predominant histologic pattern was a not otherwise specified pattern in 75%. At the time of treatment initiation most patients exhibited nephrotic range proteinuria (4.5 ± 3.6 g/d) [22]. The relapse rate in those patients achieving remission was 52% [22] and 76% [21] during a follow-up time of 12–14 months.

Larger cohorts reporting on MMF efficacy in steroid-resistance showed lower overall response rates. In a prospective study by Cattran and coworkers 33.3% of patients achieved remission (0 complete and 6 partial). Patients received a maximum dose of MMF 1 g b.i.d. and prednisone in reducing steps (initial 0.25 mg/kg body weight). In general, treatment was well tolerated with one mild gastrointestinal symptom as predominant side effect and herpes zoster in one patient [24]. Medrano et al. reported an even lower response rate with 14.8% of their patients having remission during follow-up (0 complete and 4 partial). Notably, patients in the latter study were resistant towards CSA (trough level: 150–200 ng/mL). All patients had nephrotic range proteinuria when MMF (target dose 2 g/d) was initiated. Among the side effects, dose-dependent gastrointestinal symptoms were most frequent (33.3%), whereas other adverse events may be related to the continuation of CSA treatment (gingival hyperplasia, acute renal toxicity, and worsening of hypertension) [25]. Two prospective studies from Germany highlighted that chlorambucil is an effective immunosuppressive measure in SR FSGS. While Risler et al. showed a reduction in proteinuria from 3.4 ± 4.9 to 2.3 ± 1.1 g/d in 24 subjects during a follow-up time of 36 months [19], 15 out of 23 achieved remission in another study (4 complete and 11 partial, 65.2%) following treatment with prednisolone (1.5 mg/kg per day) and chlorambucil (0.1 to 0.4 mg/kg per day) [20].

Several articles reported on the use of extracorporeal measures, either plasma exchange or immunoadsorption. In the larger cohorts, high remission rates as well as significant reduction in proteinuria were reported in two studies from Japan including patients with persistent nephrotic syndrome (12/17, 8 complete and 4 partial) as well as reduction of proteinuria from 7.24 ± 3.58 g/d to 2.56 ± 2 g/d using LDL-apheresis (twice a week for 3 weeks in total; total volume 3-4 liters; concomitant treatment not stated) [26, 27] and from Saudi Arabia (8/11, 6 complete and 2 partial) using plasma exchange (5 daily consecutive sessions, followed by twice weekly for 2 weeks, then once a week for two weeks, every two weeks for four weeks, and finally four monthly sessions; total of 17 sessions), alongside oral prednisolone (1 mg/kg body weight for two months) and six pulses of monthly cyclophosphamide (5–10 mg/kg body weight) [28]. In contrast, one study from Austria (using either protein A or immunoglobulin G immunoadsorption, five sessions within two weeks, which was repeated when ineffective) [29] and one from the USA using plasma exchange (six sessions with 1.5 plasma volume exchanged over two weeks) [30] revealed a low remission rate after addition of extracorporeal treatment (20 and 25%, resp., with a complete remission in the former and two partial remissions in the latter study). Sirolimus was tested in a prospective open-label trial including a majority of patients having nephrotic syndrome (76%) despite 3 months of prednisone therapy. In contrast to the experience in SD patients, remission was achieved in a majority of patients (57.1%) with four and eight subjects having complete and partial remission. However, no initial dosage of sirolimus and no respective trough level were given by the authors. Abdominal pain was the most frequent side effect, followed by flu-like symptoms in two and oral ulcers in one patient [31]. In an observational trial, Hogan et al. reported a response rate of 43.8% following ACTH (80 units twice weekly as subcutaneous injections) treatment in SR patients. Of the responders, 2 achieved complete (both with tip lesion) and 2 partial remission (one with tip and one with a not otherwise specified lesion) [23]. Fernandez-Fresnedo and colleagues retrospectively collected data on RTX-treated patients. They found partial remission in two (both having a not otherwise specified lesion on renal histology) out of eight patients treated with RTX, while one patient had a transient decline of proteinuria twice immediately after initiation of treatment. One patient with partial response received eight consecutive weekly infusions (a dose of 375 mg/m^2^), while the other showing response had four weekly infusions followed by two more infusions after six months. All others with no or transient response were treated with four consecutive weekly RTX infusions [32]. More efficacy data are needed for other measures, such as galactose (0.2 mg/kg twice a day, maximum dose 15 g) [33], which was tested in a recent trial. Three out of seven patients (2 with subnephrotic proteinuria) showed a partial response, while the others did not respond to galactose. In contrast, the preliminary trial performed by Trachtman and colleagues did not support the use of adalimumab (24 mg/m^2^, maximum dose 40 mg fortnightly as a subcutaneous injection), a monoclonal antibody targeting tumor necrosis factor-*α*, in the treatment of SR FSGS, since all patients recruited failed to show a response [33]. The respective results are summarized in Table 3.

## 4. Discussion

The aim of this systematic review was to summarize the progress related to treatment strategies in adult FSGS over the past two decades. Clearly, we found most reports including patients with difficult-to-treat disease forms, namely, SR FSGS, indicating the high unmet need in effective immunosuppressive measures in this subgroup of patients.

Several studies have elucidated nonimmunosuppressive effects of treatment options, including calcineurin inhibitors and rituximab. It was shown that the antiproteinuric effect of CSA may be related to stabilization of the actin cytoskeleton in podocytes rather than inhibition of the nuclear factor of activated T-cells (NFAT) pathway. CSA was capable of blocking the calcineurin-dependent degradation of synaptopodin, which colocalizes with 14-3-3ß in the adult mouse kidney. Preservation of this interaction led to protection from cathepsin L-mediated degradation. Furthermore, it was demonstrated that lipopolysaccharide- (LPS-) induced proteinuria was reduced in those severe combined immunodeficiency (SCID) mice receiving CSA treatment [36]. Stabilization of the actin cytoskeleton has also been demonstrated for rituximab. Despite its effects on CD20 bearing cells, rituximab was capable of preventing sphingomyelin-phosphodiesterase-acid-like-3b (SMPDL-3b) and acid sphingomyelinase (ASMase) downregulation. Overexpression of SMPDL-3b or treatment with rituximab of a human podocyte cell culture could prevent podocyte apoptosis or disruption of the actin cytoskeleton induced by sera of patients with recurrent FSGS after kidney transplantation. Moreover, the incidence of recurrent nephrotic range proteinuria and decline in estimated glomerular filtration rate 3 and 6 months following kidney transplantation were lower in the rituximab-treated patients [37]. Given these observations, stabilization of the actin cytoskeleton as a potential nonimmunosuppressive effect has emerged as an explanation of calcineurin inhibitor and rituximab efficacy in the treatment of proteinuric glomerular disease including FSGS.

Patients with primary FSGS should receive RAAS-blockade, either ACE-inhibitor treatment or angiotensin receptor blockade if contraindications are ruled out. An analysis of patients with FSGS highlighted that the use of RAAS-blockade was associated with better renal survival and a slower progression of chronic kidney disease in univariate analysis. Although this association became nonsignificant in multivariate analysis [38], experience from other entities clearly supports its role in the long-term treatment. As a first-line immunosuppressive treatment strategy, daily oral prednisolone has emerged as a suitable therapeutic option with good remission rates. Since spontaneous remission is rare in primary FSGS [2], treatment should be initiated after the result of the renal biopsy is retrieved especially in those with high risk of progression and secondary forms are excluded. The relapse rate following cessation of therapy may be around 30–70%, depending on the length of follow-up. In cases with relative or absolute contraindication towards steroids such as psychosis, severe obesity, or impaired glucose tolerance, other immunosuppressive measures may be used. Remission rates have been shown to be similar for tacrolimus, CPA, or MMF-treated patients.

In those patients with SD/MR disease, experience is limited to single reports. Calcineurin inhibitors (CSA) have shown good remission rates. One limitation is the relative small number of adult patients included in these studies. Most experience in this setting has been gathered for MMF treatment. Again, efficacy is comparable with those treated with CSA. Rituximab has shown encouraging effects in SD/MR adult FSGS as has been shown in the maintenance of remission. We have reported a small case series [39] which again highlighted its potential. Other measures have been tested in single center studies, and there is no recommendation to use ACTH (4 out of 5 patients resistant) [23] as well as sirolimus (overall increase of proteinuria after treatment was commenced) [15] in this setting.

Most experience has been published in patients with steroid-refractoriness. In this form, calcineurin inhibitors have shown good efficacy in diverse ethnicities with a high relapse rate after cessation in those with an initial response. Most patients receiving MMF/MPA on top of their steroid treatment did not achieve remission. Thus, the recommendation in the latest KDIGO glomerulonephritis guidelines in the setting of steroid-refractoriness and contraindication towards calcineurin inhibitors [12] is not supported by our systematic review. Interestingly, no study reported efficacy of CPA in more than five patients over the last two decades. Again, in the KDIGO glomerulonephritis guidelines, CPA should be considered in SR nephrotic syndrome in children (mainly FSGS as entity) [12]. In patients with rapid-progressive deterioration of renal function and idiopathic membranous nephropathy, the addition of an alkylating agent (in this case chlorambucil) was superior to CSA or supportive treatment in abrogating further decline in renal function [40]. This may highlight that, in patients with a refractory disease course, alkylating agents may be of particular interest in FSGS as well. In line with this assumption, two studies from Germany showed either good remission rates (4 and 11 achieved complete and partial remission and eight no remission) or a decline in proteinuria following initiation of chlorambucil treatment [19, 20]. Fernandez-Fresnedo et al. published results from the Spanish GLOSEN registry on the use of rituximab in SR FSGS, highlighting persisting partial remission in two out of eight and a transient partial response in one subject. The others did not show any benefit following rituximab treatment [32]. Conflicting results have been observed and published related to extracorporeal treatment forms (either immunoadsorption or plasma exchange) in the treatment of naïve kidney FSGS. However, addition of immunoadsorption or plasma exchange may be considered in patients unresponsive to several immunosuppressive measures, given its theoretical effects on removing the “circulatory factor(s).” The total number of patients treated with other strategies such as sirolimus, ACTH, adalimumab, or galactose is low [15, 31, 33]. Galactose may be an agent of interest, since it may be effective in some cases and adverse events attributable to its treatment may be absent [33].

In consideration of additional immunosuppressive agents weighing pros and cons taking into account side effects is pivotal. Most reports included herein did not report adverse events. In those recording side effects, most complications have been non-life-threatening. Nevertheless, treating physicians need to be aware of potential side effects, especially when prescribing novel agents such as rituximab. No concrete life-threatening side effects have been reported in a recent meta-analysis from our institute when treatment of idiopathic nephrotic syndrome was analyzed [41]. However, in other autoimmune diseases, such as ANCA-associated vasculitis, B-cell depletion may exhibit a more severe side effect spectrum with fatal infectious complications such as* Pneumocystis jirovecii* pneumonia or septic conditions [42].

Several limitations need to be taken into account related to our work: (i) a heterogeneity in patients included in the different studies, in terms of pretreatment and proteinuria at the time of treatment initiation; (ii) different definitions used to define steroid-dependence or steroid-resistance (as most studies have not used current KDIGO guidelines); (iii) unclear histopathologic lesions according to the Columbia classification; (iv) lack of clinical variables of interest, such as serum albumin (± increase following treatment response); (v) nonuniform reporting of outcomes (i.e., only proteinuria response and not rates of either complete or partial remission). Thus, we would encourage researchers in the field to add all these variables in future trials in nephrotic syndrome in general. Clearly, our review highlights the need that more effort in general is necessary to improve patient care (outcome) in FSGS.

## 5. Conclusion

The identification of the “circulatory factor(s)” is of importance, since we may be able to tailor immunosuppressive agents to its presence and the intensity of removal strategies (i.e., immunoadsorption) may be adapted towards its blood concentration. Despite the improved understanding of podocyte biology with identification of several nonimmunosuppressive targets of immunosuppressive agents used, the ideal treatment strategy has not been discovered in SD/MR or SR patients. In first-line treatment, daily oral prednisolone is a valuable option, whereas calcineurin inhibitors may be considered in those patients with SD/MR or steroid-resistance. Other options in the first case are rituximab or MMF/MPA, whereas both agents may not be effective in steroid-resistance. This patient group may benefit from early switch to alkylating agents, either CPA or chlorambucil, and the addition of extracorporeal options may be considered. Clearly, more studies, favorable in a prospective manner, may pave the way to improve patient care in primary FSGS.

## Figures and Tables

**Table 1:** 

First author	Year	Design	Study	Country	PRED	TAC	CSA	MMF	AZA	No	CR	PR	NR	PR (BL)	PR (FU)	RL (%)	FU (m)
Rydel [5]	1995	Retro	Cohort	USA	1	0	0	0	0	30	12	3	15	13.6 ± 10		67	62
Ponticelli [6]	1999	Retro	Cohort	Italy	1	0	0	0	0	53	21	10	22			25.8	
Choi [7]	2002	Retro	Cohort	USA	0	0	0	1	0	10	3	2	5	4.5 ± 3.1	2.6 ± 2.9		7.9
Duncan [8]	2004	Pro	Cohort	UK	0	1	0	0	0	6	0	6	0	11 ± 4.5	2.8 ± 2.5	0	12.8
Goumenos [9]	2006	Retro	Cohort	UK/Greece	1	0	0	0	0	8		5	3				
Goumenos [9]	2006	Retro	Cohort	UK/Greece	1	0	1	0	0	7		6	1				
Goumenos [9]	2006	Retro	Cohort	UK/Greece	1	0	0	0	1	5		4	1				
Senthil Nayagam [10]	2008	Pro	RCT	India	1	0	0	1	0	17	10	2	5			33.3	15.3
Senthil Nayagam [10]	2008	Pro	RCT	India	1	0	0	0	0	16	9	2	5			27.3	16.2
Jafry [11]	2012	Retro	Cohort	Egypt	1	0	0	0	0	79	36	4	39	6 ± 4.4	4.6 ± 5.1	35	26

AZA: azathioprine, CR: complete remission, CSA: cyclosporine A, FU: follow-up, MMF: mycophenolate mofetil, No: number, NR: no remission, PRED: prednisone/prednisolone, PR: partial remission, PR (BL): proteinuria baseline, PR (FU): proteinuria follow-up, Pro: prospective, Retro: retrospective, RL: relapse, and TAC: tacrolimus.

**Table 2:** 

First author	Year	Design	Study	Country	CSA	MMF	RTX	SIR	ACTH	No	CR	PR	NR	PR (BL)	PR (FU)	RL (%)	FU (m)
Lee [13]	1995	Pro	Observational	Korea	1	0	0	0	0	5	4	1	0				18
Cho [15]	2007	Pro	Clinical trial	USA	0	0	0	1	0	6	0	0	6	8.4 ± 6	12.3 ± 5.8		8
Dimkovic [14]	2009	Pro	Cohort	Serbia	0	1	0	0	0	10	3	5	2	5.1	1.9		
Hogan [23]	2013	Pro	Observational	USA	0	0	0	0	1	6	0	2	4	7.7 ± 6.2	8 ± 9.7		
Ruggenenti [16]	2014	Pro	Observational	Italy	0	0	1	0	0	8				0.3	0.2	37.5	12

ACTH: adrenocorticotropic hormone, CR: complete remission, CSA: cyclosporine A, FU: follow-up, MMF: mycophenolate mofetil, No: number, NR: no remission, PR: partial remission, PR (BL): proteinuria baseline, PR (FU): proteinuria follow-up, Pro: prospective, Retro: retrospective, RL: relapse, RTX: rituximab, and SIR: sirolimus.

**Table 3:** 

First author	Year	Design	Study	Country	TAC	CSA	MMF/MPA	RTX	CA	ET	SIR	ACTH	GAL	ADA	No	CR	PR	NR	PR (BL)	PR (FU)	RL (%)	FU (m)
Ittel [18]	1995	Retro	Cohort	Germany	0	1	0	0	0	0	0	0	0	0	7	1	3	3	13.7 ± 3.8	4.7 ± 0.4		6
Risler [19]	1996	Pro	RCT	Germany	0	1	0	0	0	0	0	0	0	0	23				5.4 ± 5.2	2.5 ± 1.8		36
Risler [19]	1996	Pro	RCT	Germany	0	0	0	0	1	0	0	0	0	0	24				3.4 ± 4.9	2.3 ± 1.1		36
Yokoyama [27]	1998	Retro	Cohort	Japan	0	0	0	0	0	1	0	0	0	0	14				7.2 ± 3.6	2.6 ± 2.0		
Mitwalli [28]	1998	Retro	Cohort	Saudi Arabia	0	0	0	0	0	1	0	0	0	0	11	6	2	3	5.3 ± 1.2	1.4 ± 0.6		27.5
Haas [29]	1998	Retro	Cohort	Austria	0	0	0	0	0	1	0	0	0	0	5	1	0	4	13.6 ± 8.9	11.9 ± 10.6		
Feld [30]	1998	Retro	Cohort	USA	0	0	0	0	0	1	0	0	0	0	8	0	2	6			0	24
Cattran [17]	1999	Pro	RCT	North America	0	1	0	0	0	0	0	0	0	0	26	3	15	8	6.9 ± 3.3		61	24
Ponticelli [6]	1999	Retro	Cohort	Italy	0	1	0	0	0	0	0	0	0	0	9	0	7	2				
Muso [26]	2001	Retro	Cohort	Japan	0	0	0	0	0	1	0	0	0	0	17	8	4	5	6.2 ± 3.3	2.7 ± 2.7		
Segarra [21]	2002	Retro	Cohort	Spain	1	0	0	0	0	0	0	0	0	0	25	10	8	7	10.3 ± 9.5	2.6 ± 3.2	76	12
Heering [20]	2004	Pro	Observational	Germany	0	1	0	0	0	0	0	0	0	0	34	8	13	13	5.5 ± 2.6			
Heering [20]	2004	Pro	Observational	Germany	0	0	0	0	1	0	0	0	0	0	23	4	11	8	4.2 ± 0.6			
Cattran [24]	2004	Pro	Observational	USA	0	0	1	0	0	0	0	0	0	0	18	0	6	12	9.1 ± 5.2	6.8 ± 6.1		
Tumlin [31]	2006	Pro	Clinical trial	USA	0	0	0	0	0	0	1	0	0	0	21	4	8	9	8 ± 1.2	3.9 ± 0.7		6
Fernandez-Fresnedo [32]	2009	Retro	Cohort	Spain	0	0	0	1	0	0	0	0	0	0	8	0	3	5	14 ± 4.4	11.3 ± 4.2		12
Li [34]	2009	Pro	Observational	China	1	0	0	0	0	0	0	0	0	0	7	3	1	3	7	1.4	25	12
Medrano [25]	2011	Retro	Cohort	Spain	0	0	1	0	0	0	0	0	0	0	27	0	4	23	7.7 ± 3.9	6.0 ± 4.1		12
Hogan [23]	2013	Pro	Observational	USA	0	0	0	0	0	0	0	1	0	0	16	2	2	12	6.3 ± 6	4.1 ± 4.8	14	
Fan [35]	2013	Pro	Observational	China	1	0	0	0	0	0	0	0	0	0	7	3	3	1			17	12
Ramachandran [22]	2014	Pro	Observational	India	1	0	0	0	0	0	0	0	0	0	44	17	6	21	4.5 ± 3.6	0.5 ± 0.5	52	14
Trachtman [33]	2015	Pro	RCT	USA	0	0	0	0	0	0	0	0	1	0	7	0	3	4	5.4 ± 5	6.7 ± 3.5		6
Trachtman [33]	2015	Pro	RCT	USA	0	0	0	0	0	0	0	0	0	1	6	0	0	6	12.2 ± 17	7.6 ± 10.5		6

ACTH: adrenocorticotropic hormone, ADA: adalimumab, CA: chlorambucil, CR: complete remission, CSA: cyclosporine A, ET: extracorporeal treatment, FU: follow-up, GAL: galactose, MMF: mycophenolate mofetil, MPA: mycophenolic acid, No: number, NR: no remission, PR: partial remission, PR (BL): proteinuria baseline, PR (FU): proteinuria follow-up, Pro: prospective, Retro: retrospective, RL: relapse, RTX: rituximab, SIR: sirolimus, and TAC: tacrolimus.
